# Prevalence of intestinal parasitic infections and associated risk factors among pregnant women attending antenatal care center at Felege Hiwot Referral Hospital, northwest Ethiopia

**DOI:** 10.1186/s12879-016-1859-6

**Published:** 2016-09-30

**Authors:** Adane Derso, Endalkachew Nibret, Abaineh Munshea

**Affiliations:** Department of Biology, College of Science, Bahir Dar University, P.O.Box 79, Bahir Dar, Ethiopia

**Keywords:** Intestinal parasites, Pregnant women, Bahir Dar, Ethiopia

## Abstract

**Background:**

Parasitic infections affect tens of millions of pregnant women worldwide, and directly or indirectly lead to a spectrum of adverse maternal and fetal/placental effects. The objective of this study was to assess the prevalence of intestinal parasite infections and associated risk factors among pregnant women attending antenatal care center in Felege Hiwot Referral Hospital, Bahir Dar city, northwest Ethiopia.

**Methods:**

A cross-sectional hospital based study was conducted from November 2013 to January 2014 among 384 pregnant women. Stool samples were examined for the presence of trophozoites, cysts, oocysts, and ova using direct, formal-ether sedimentation, and modified Ziehl-Neelsen techniques.

**Results:**

An overall prevalence of 31.5 % intestinal parasite infections was recorded. Eight different species of intestinal parasites were found: two protozoan and six helminth species. The highest prevalence was due to *Giardia lamblia* (13.3 %) followed by *Entamoeba histolytica*/*dispar* (7.8 %), hookworm (5.5 %), *Ascaris lumbricoides* (2.9 %), *Schistosoma mansoni* (2.9 %), *Strongyloides stercoralis* (1.6 %), *Taenia* spp. (0.8 %), and *Hymenolepis nana* (0.3 %).

**Conclusions:**

A relatively high prevalence of intestinal parasite infections was observed among pregnant women. Routine stool examination and provision of health education are required for early medical intervention that would affect the pregnant mothers and their foetuses.

**Electronic supplementary material:**

The online version of this article (doi:10.1186/s12879-016-1859-6) contains supplementary material, which is available to authorized users.

## Background

Intestinal parasitic infections constitute a global health burden causing clinical morbidity in 450 million people, many of these are women of reproductive age and children in developing countries [1]. Elevated intestinal parasitic infections have been recorded in developing countries because of poverty, low literacy rate, lack of safe drinking water, poor hygiene, malnutrition and hot and humid tropical climate [2, 3].

Tens of millions of pregnant women as one of the segments of the community are affected by parasitic infections which directly or indirectly lead to a spectrum of adverse maternal and fetal/placental effects. Pregnant women often experience more severe infections than their non-pregnant counterparts [4]. Parasitic infection could occur at any stage of the three trimesters during pregnancy, but infection during the first trimester is associated with more severe fetal and placental consequences than those occurring later in pregnancy. Furthermore, the infection becomes more severe in women who are pregnant for the first time (primigravida) compared with other gravidae [5].

Intestinal parasitic infections (IPIs) are a major concern, mostly in developing countries, particularly in sub-Saharan Africa (SSA) [6]. Ethiopia has one of the lowest quality drinking water supply and latrine coverage in the world [7] and because of this and other risk factors, intestinal parasitic infections are the second most predominant causes of outpatient morbidity in the country. Several previous studies about parasitic infection conducted in Ethiopia have mainly focused on preschool children and school children [8–10]. The current study was necessitated to assess the prevalence of intestinal parasitic infection which is not well addressed among pregnant women of the country in general and those of Bahir Dar city in particular.

## Methods

### Study design and study area

A cross-sectional hospital based study was conducted from October 2013 to January 2014 so as to determine prevalence of intestinal parasitic infections and associated risk factors among pregnant women attending antenatal center at Felege Hiwot Referral Hospital, Bahir Dar, Ethiopia. Bahir Dar is the capital of the Amhara National Regional State in the Federal Democratic Republic of Ethiopia. It is located at 11°36’ latitude N and 37°23’ longitude E in north-western part of the country with an elevation of 1800 m above sea level and it is about 570 kms away from Addis Ababa [Bahir Dar City Administration Office, General information about the city, unpublished]. According to Amhara Bureau of Finance and Economic Development (BOFED), population of Bahir Dar city was estimated to be 284,020. Among these, 149,202 of them are females [11]. The city has one referral hospital (Felege Hiwot Hospital), 10 health centers, 10 health posts and one family guidance association clinic and other private clinics [Bahir Dar City Health Bureau, Health infrastructure in Bahir Dar City, unpublished].

### Sample size determination

In the estimation of the sample size, statistical formula for sample size calculation and previous work for prevalence of infectious diseases was considered as a basis [12]. Since prevalence of intestinal parasitic diseases in the study area was not known, the sample size of the proposed study was calculated using 50 % prevalence, and a total of 384 pregnant women included in the study.

### Sampling techniques

After obtaining written consent, a random sampling method was employed to include 384 study participants. Before sample collection, a brief explanation of the aims of study was given to volunteers and information was collected using a pre-structured questionnaire which contained questions related to socio-demographic characteristics, behavioral habits, and environmental conditions.

Each participant was provided with labeled screw capped stool container and informed on how to collect stool samples. Parts of the collected stool specimens were checked for trophozoites of protozoans and the remaining parts were preserved with 10 % formalin. The collected stool samples were transported to Bahir Dar University Biomedical and Microbiology Laboratory and processed following the standard procedure using formol-ether concentration method for the detection of helminthes and for some of the protozoans [13] and Modified Ziehl-Neelsen technique for detection of *Cryptosporidium* spp [14].

### Source and study population

All pregnant women who were attending antenatal clinic at Felege Hiwot Referral Hospital were considered as a source population while those who visited the same during sample collection period were considered as a study population.

### Inclusion and exclusion criteria

Pregnant women who attended antenatal clinic at the study period and those who were willing to provide stool samples were included in the study and those who were not volunteers to give stool samples were excluded from the study.

### Data analysis

Information recorded in the questionnaire and results collected from laboratory were checked for completeness and consistency and then coded and entered into computer. The compiled data were analyzed using Statistical Package for the Social Sciences (SPSS version 20.0). The magnitude of associations between intestinal parasitic infection and possible risk factors was determined using logistic regression and described in terms of odds ratio (OR) at 95 % CI. After univariate logistic regression analysis, all variables with a *p*-value less than or equal to 0.3 were entered into multivariate logistic regression to identify independent risk factors for the occurrence of intestinal parasitic infection among pregnant women [15]. The raw data used for the statistical analysis of this study are shown in Additional file 1.

## Results

### Socio-demographic characteristics of study subjects

A total of 384 pregnant women were included in the study and a 100 % response rate was obtained in filling out the questionnaires. The mean age of the study participants was 27.1 ± 4.65 year. The age of participants ranged from 18 to 44 years. Of the total study subjects, 358 (93.2 %) were married, 21 (5.5 %) were single and the rest 5 (1.3 %) were divorced. One hundred twenty two (31.8 %) women attended college or university and 116 (30.2 %) attended high school. One hundred three (26.8 %) could write and read. Forty three (11.2 %) women were illiterate. Out of 384 participants, 44 (11.5 %) were rural residents and the rest 340 (88.5 %) were urban dwellers (Table 1).

**Table 1:** Socio-demographic characteristics of pregnant women in Felege Hiwot Referral Hospital, Bahir Dar, Ethiopia (October 2013 - January 2014)

Socio-demographic characteristics	Number of women	Percent
Age (year)		
15 – 19	5	1.3
20 - 24	108	28.1
25 - 29	176	45.8
30 - 34	61	15.9
35 - 39	27	7.0
40 - 44	7	1.8
Marital status		
Married	358	93.2
Single	21	5.5
Divorced	5	1.3
Education		
Illiterate	43	11.2
Writing and reading	103	26.8
High school	116	30.2
College/University and above	122	31.8
Residence		
Rural	44	11.5
Urban	340	88.5
Occupation		
Governmental employee	111	28.9
Private worker	83	21.6
Housewives	162	42.2
Daily laborers	17	4.4
Others	11	2.9

### Medical history of study subjects

Two hundred nine (54.4 %) pregnant women were in the third trimester (gestational age greater than 28 weeks), while 136 (35.4 %) were in their second trimester (between 13 and 28 weeks of gestation). Thirty nine (10.2 %) of the participants were in the first trimester (gestational age less than 13 weeks). Nearly half (43.5 %) of pregnant women were without a previous pregnancy. One hundred eighteen (30.7 %) participants had one child and the remaining 99 (25.8 %) women were pregnant for more than two times (Table 2).

**Table 2:** Univariate analysis of intestinal parasitic infection across socio-demographic and medical history characteristics of pregnant women in Felege Hiwot Referral Hospital, Bahir Dar, Ethiopia (October, 2013 - January, 2014)

Characteristics	No examined (%)	Intestinal parasites	COR (95 % CI)	*P*-value
		Positive (%)		
Educational level				0.44
Illiterate	43 (11.2)	18 (41.9)	1.65 (0.81 – 3.39)	
Writing and reading	103 (26.8)	33 (32.0)	1.08 (0.62 – 1.91)	
High school	116 (30.2)	33 (28.4)	0.91 (0.52 – 1.59)	
College/University and above	122 (31.8)	37 (30.3)	1	
Marital status				0.39
Married	358 (93.2)	116 (33.2)	1.92 (0.21 – 17.35)	
Single	21 (5.5)	4 (17.1)	0.94 (.08 – 10.88)	
Divorced	5 (1.3)	1 (16.7)	1	
Monthly income (Ethiopian Birr)				
Less than 500	74 (19.3)	29 (39.2)	1.43 (0.83– 2.47)	0.20
500 - 1000	88 (22.9)	23 (26.1)	0.77 (0.45 –1.37)	
Above 1000	222 (57.8)	69 (31.1)	1	
Occupation				0.89
Governmental employee	111 (28.9)	36 (32.4)	1	
Private work	83 (21.6)	24 (28.9)	0.85 (0.46 – 1.57)	
Housewives	162 (42.2)	54 (33.3)	1.04 (0.62 – 1.74)	
Daily laborers	17 (4.4)	4 (23.5)	0.64 (0.20 – 2.11)	
Others	11 (2.9)	3 (27.3)	0.80 (0.20 – 3.12	
Residence				0.70
Urban	340 (88.5)	106 (31.2)	1	
Rural	44 (11.5)	15 (34.1)	1.14 (0.59 – 2.22)	
Parity				0.62
Primegravidae	167 (43.5)	57 (34.1)	1.25 (0.73 – 2.14)	
Bigravidae	118 (30.7)	35 (29.7)	1.02 (0.57 – 1.83)	
Multigravidae	99 (25.8)	29 (29.3)	1	
Trimester				0.62
1st	39 (10.2)	11 (28.2)	0.91 (0.43 – 1.94)	
2nd	136 (35.4)	47 (34.6)	1.22 (0.77 – 1.94)	
3rd	209 (54.4)	63 (30.1)	1	
Age (year)				0.64
15 - 19	5 (1.3)	1 (20)	0.63 (0.04 – 9.65)	
20 - 24	108 (28.1)	33 (30.6)	1.10 (0.20 – 5.96)	
25 - 29	176 (45.8)	53 (30.1)	1.08 (0.20 – 5.73)	
30 - 34	61 (15.9)	25 (41.0)	1.74 (0.31 – 9.67)	
35 - 39	27 (7.0)	7 (25.9)	0.88 (0.14 – 5.58)	
40 - 44	7 (1.8)	2 (28.6)	1	

#### Prevalence of intestinal parasite infection across environmental and sanitary conditions of study participants

The results in Table 3 indicate the prevalence of intestinal parasitic infections with respective to environmental and sanitary conditions of the study participants. All the potential risk factors studied did not show statistical significant associations with intestinal parasitic infections (*p* > 0.05). Nevertheless, for example, in relation to behavioral factors, 31.3 % (115/367) of studied pregnant women who had the habit of washing hands before eating were infected with intestinal parasites, in comparison to 35.3 % (6/17) of women who did not wash their hands before meal. Additionally, 30.9 % (116/376) were infected in group practicing hand washing after toilet, in comparison to 62.5 % (5/8) women who did not wash their hands after toilet. With regard to feeding habits, 33.8 % (77/228) of participants who ate raw/unwashed vegetables were infected, compared to 28.2 % (44/156) of women who did not eat raw/unwashed vegetables. Regarding sanitary facility, 31.2 % (116/372) of women who possessed toilet were infected with intestinal parasites, in comparison to 41.7 % (5/12) who did not possess toilet in their houses.

**Table 3:** Univariate analysis of intestinal parasitic infections in relation across hygiene and environmental factors of pregnant women in Felege Hiwot Referral Hospital, Bahir Dar (November 2013 – January 2014)

Characteristics	No examined (%)	Intestinal parasites	COR (95 % CI)	*P*-value
		Positive (%)		
Hand washing before meal				0.73
Yes	367 (95.6)	115 (31.3)	1	
No	17 (4.4)	6 (35.3)	1.19 (0.43 – 3.31)	
Raw/unwashed vegetables				0.25
Yes	228 (59.4)	77 (33.8)	1.29 (0.83 - 2.02)	
No	156 (40.6)	44 (28.2)	1	
Hand washing after toilet				0.07
Yes	376 (97.9)	116 (30.9)	1	
No	8 (2.1)	5 (62.5)	3.74 (0.88 – 15.89)	
Toilet presence				0.45
Yes	372 (96.9)	116 (31.2)	1	
No	12 (3.1)	5 (41.7)	1.58 (0.49 – 5.07)	
Soil contact				0.35
Yes	87 (22.7)	31 (35.6)	1.27 (0.77 – 2.11)	
No	297 (77.3)	90 (30.3)	1	
Water drinking source				0.93
Tap water	358 (93.2)	113 (31.6)	1	
Well	26 (6.8)	8 (30.8)	0.96 (0.41 – 2.28)	
Soil eating				0.55
Yes	30 (7.8)	8 (26.7)	0.78 (0.34 – 1.79)	
No	354 (92.2)	113 (31.9)	1	

### Overall and species specific prevalence of intestinal parasitic infection

Intestinal parasitic infection was observed in 121 pregnant women and the overall prevalence was 31.5 %; out of which 81 cases (21.1 %) were caused by protozoans whereas 53 cases (13.8 %) were caused by helminthes. From those pregnant women who were infected, 13 cases were due to simultaneous infection by two different intestinal parasites. In this study, eight different species of intestinal parasites were identified, two protozoans, and six helminths species. *Giardia lamblia* was the most common protozoan parasite (13.3 %) followed by *E.histolytica*/*dispar* (7.8 %). Besides, helminthes identified in this study were *Ascaris lumbricoides*, hookworm, *Strongyloides stercoralis*, *Schistosoma mansoni*, *Taenia* species and *Hymenolepis nana* with their respective prevalence of 2.9 %, 5.5 %, 1.6 %, 2.9 %, 0.8 %, and 0.3 %.

#### Univariate and multivariate analysis of risk factors in relation to prevalence of intestinal parasitic infections

The univariate analysis results across socio-demographic, medical history, environmental, and sanitary factors are presented as crude odds ratios (COR) with 95 % confidence intervals (Tables 2 and 3). However, none of socio-demographic, environmental, and behavioral factors were found to be independent explanatory variables in multivariate logistic regression analysis.

The univariate analysis (Table 2) of intestinal parasitic infection across socio-demographic and medical history revealed that the odds of infection with intestinal parasites were almost two times higher in illiterate than in those pregnant women whose educational level was at college or university (COR = 1.65, *p* = 0.44). It was also found that the odds of being infected with intestinal parasites in those who were married were almost two times higher (COR = 1.92, *p* = 0.39) than in those who were divorced or single. The odds of intestinal parasitic infection were increased by 43 % in those whose monthly income was below 500 Ethiopian Birr compared to those whose monthly income was above 1000 Ethiopian Birr. Regarding residential area of the women, the odds for intestinal parasitic infection were increased by 14 % in those who lived in rural areas compared to those who lived in urban areas. The odds for intestinal parasitic infection were increased by 25 % in primegravidae women compared to multigravidae women. The odds of intestinal parasitic infection were increased by 22 % in the second trimester women compared to those in their third trimester of pregnancy. Regarding the age of the women, the odds for intestinal parasitic infection were almost two times higher (COR = 1.74, *p* = 0.64) in those whose age was between 30 and 34 years than in those whose age was between 40 and 44 years.

The output of statistical analysis provided in Table 3 shows intestinal parasitic infections across hygiene and environmental factors. As hand washing practice before meal was decreased by one, the odds of intestinal parasitic infection were increased by 19 %. As eating raw/unwashed vegetables was increased by one at every meal, the odds of intestinal parasitic infection were increased by 29 %. Additionally, the statistical analysis showed that the odds of infection were about four times higher (COR = 3.74, *p* = 0.07) in pregnant women who had no habit of hand washing after toilet than those who had the habit of washing their hands after toilet. It was showed that the odds of infection with intestinal parasites were almost two times higher in pregnant women who had no toilet (COR = 1.58, *p* = 0.45) in comparison to women who had access to toilet. It was also found that as the soil contact was increased by one, the odds for intestinal parasitic infection were increased by 27 %.

## Discussion

Estimating the burden of a disease requires adequate epidemiological information [16]. The present study was therefore focused on determining the prevalence of intestinal infections and associated risk factors among pregnant women. Surprisingly, high prevalence of intestinal parasites (31.5 %) was recorded among pregnant women attending Felege Hiwot Referral Hospital, Bahir Dar city, Ethiopia. Such a relatively high prevalence of intestinal parasitic infection observed in the present study might be because of environmental pollution of parasite cysts, ova, and larvae and this could be one of the reasons why there is permanent transmission of parasites among humans in endemic regions. Intestinal protozoa are known to be transmitted by the fecal-oral route and their transmission involves the ingestion of food or water contaminated with cysts. The ova of soil-transmitted helminthes such as *A. lumbricoides*, *H. nana* could be ingested from contaminated fingers, water, food or soil. Hookworm and *S. stercoralis* infections are acquired when the larvae penetrate exposed human skin from contaminated soil. *S. mansoni* infection is acquired when the larval stage, cercaria, penetrates the human body from contaminated water bodies. In case of *Taenia* spp, consumption of raw or inadequately cooked, infected beef (in case of *T. saginata*) or pork (in case of *T. solium*) introduces the larvae into the human intestinal tract where they mature into adult worms [17].

The prevalence of intestinal parasites identified in the present study (31.5 %) was higher than 14.3 % which was reported among pregnant women at Azezo Health Center of Gondar town, northwest Ethiopia [18] and those of 17.6 % and 13 % reported from Ghana [19] and Senegal [20], respectively. However, our finding was lower than the findings of other similar types of studies reported from Nigeria (43.4 %) [21], Kenya (76.2 %) [22], Gabon (65 %) [23], and Venezuela (73.9 %) [24]. Difference in findings among various studies could be explained by variations in geography, socio-economic conditions, and cultural practices of the population under consideration. The specific type of study subjects, the methods employed for stool examination, and the time of study may have also contributed to the variation. Furthermore, variation in the prevalence of geophagy (soil eating) among different communities may be one of the reasons for observed varied prevalence of intestinal parasitic infections. Geophagy is thought by many as adaptive behavior to correct nutritional deficiencies, for example, iron deficiency [25]. In other study, however, it has been shown that pregnant women eat soils for varied reasons. Some pregnant women have preference for texture of soil while others for its taste [26]. Nevertheless, this soil eating habit may be a risk factor for soil-transmitted helminthic infection. For example, a study by Kawai et al. [27] showed that the prevalence of geophagy was associated with *A. lumbricoides* infection, not with hookworm and *S. stercoralis* infections. It is known that infection by most parasites decrease reproduction in animals including humans. But, a recent study among women of Tsimane, Bolivia, revealed an interesting result in which infection with *A. lumbricoides* was associated with earlier first births and shortened interbirth intervals, whereas infection with hookworm was associated with delayed first pregnancy and extended interbirth intervals [28].

Prevalence of double infections in the current study accounted 3.38 %. However, this double infection rate was relatively lower than previously reported prevalence [29]. All of the socio-demographic variables considered in the present study were found to be non-significant predictors of intestinal parasitic infections among pregnant women.

In the current finding, all environmental and sanitary risk factors do not seem to play major roles in the occurrence of intestinal parasitic infections in the studied pregnant women. However, research done in Teda town at Teda Health Center, Gondar, Ethiopia showed that the variables age, absence of toilet, and hand washing after toilet to be significantly associated with the occurrence of intestinal parasitic infections [30].

## Conclusions

The overall prevalence of intestinal parasitic infection was relatively high. The proportion of intestinal protozoal infection was higher than that of helminthic infection. *Giardia lamblia* and hookworm were the most dominant parasites from protozoans and helminthes, respectively. None of the variables included in the present study as potential risk factors showed statistical significant associations with intestinal parasitic infections.

### Limitations of the study

Compared to PCR diagnostic technique, modified Ziehl-Neelsen method is less sensitive for the detection of *Cryptosporidium* spp., and this might be one of the reasons why *Cryptosporidium* spp could not be detected in the study subjects. Moreover, light microscopy fails to differentiate between *E. histolytica* and *E. dispar* and also between *T. saginata* and *T. solium*.

## Additional file

Additional file 1: Data used for statistical analysis of the present study. Sheet1 of xls data shows the data used for statistical analysis of the present study whereas the second sheet (sheet 2) shows the summary of statistical analysis of some of the socio-demographic variables studied. (XLS 156 kb)

## Abbreviations

BOFED: Amhara Bureau of Finance and Economic Development
COR: Crude odds ratio
IPIs: Intestinal parasitic infections
OR: Odds ratio
PCR: Polymerase chain reaction
SPSS: Statistical package for the social sciences
SSA: sub-Saharan Africa
